# The Carbohydrates

**Published:** 1900-08

**Authors:** 


### The Carbohydrates.

Abel summarizes the value of carbohydrates for muscular work as follows:

1. When the organism is adapted to the digestion of starch, and there is sufficient time for its utilization, sugar has no advantage over starch as a food for muscular work except as a preventive for fatigue.
2. In small quantities, and in not too concentrated form, sugar will take the place, practically speaking, weight for weight, of starch as a food for muscular work, barring the difference in energy and in time required to digest them, sugar having here the advantage.
3. It furnishes the needed carbohydrate material to organisms that have as yet little or no power to digest starch. Thus milk sugar is part of the natural food of the infant.
4. In times of great exertion or exhausting labor, the rapidity with which it is assimilated gives it certain advantages over starch.

The value of sugar in cold climates then is apparent, for in these regions where starch food cannot be kept, sugar can be readily supplied, and, in fact, polar expeditions now ship large quantities of sugar as an article of diet. In time it may take the place of fat consumed by the inhabitants of these northern latitudes.

In tropical countries the consumption of sugar is very large, and in India it is said that the workmen must have large quantities daily with their food, or quit the job.

Milk is the only source of milk sugar, and as this liquid constitutes a large part of the dietary of the child up to two years of age, the milk sugar imparting the flavor to the milk, one can easily understand how all important it is to prohibit or curtail to a very great extent all confectionery in the diet of children, because in the infant, gorged with sweets, the appetite is palled and there will be no natural stimulus to partake of milk. Three or four ounces can be digested by the healthy adult in the twenty-four hours, without difficulty, if it is not partaken of in indigestible forms. Prof. Miller, of Berlin, states that sugar is not harmful to the teeth, although if it be allowed to cling to the teeth after eating, it may ferment, and any acidulous substances produced may then do incalculable harm to these organs. The negroes of the West Indies, who consume large quantities of sugar, have the finest teeth in the world.—*Georgia Journal of Medicine and Surgery.*

